# Research progress on acupuncture intervention for cervical spondylotic radiculopathy with Qi stagnation and blood stasis syndrome: A review

**DOI:** 10.1097/MD.0000000000041837

**Published:** 2025-04-04

**Authors:** Furong Xue, Weiguo Liu

**Affiliations:** a Department of Traditional Chinese Medicine, Affiliated Hospital of Weifang Medical University, Weifang, China; b School of Traditional Chinese Medicine, Weifang Medical University, Weifang, China.

**Keywords:** acupuncture, cervical spondylotic radiculopathy, mechanism, review

## Abstract

With improvements in living standards and lifestyle changes, the prevalence of cervical spondylotic radiculopathy (CSR) has significantly risen, severely impacting patients’ quality of life. Currently, Western medicine primarily treats CSR with oral medications and surgical interventions. However, the condition often recurs, with a high risk of side effects, failing to provide a fundamental cure. As research in traditional Chinese medicine (TCM) on this condition deepens, acupuncture has made significant progress, demonstrating notable clinical efficacy. Acupuncture has been shown to be highly effective in treating radiculopathy associated with cervical spondylopathy, characterized by its simple procedure, high safety, and diverse treatment methods, and has gained widespread recognition. This paper reviews the clinical research literature on acupuncture for CSR, summarizing acupuncture interventions and related therapies from 4 perspectives: simple acupuncture therapy, combined acupuncture and external therapy, and pathogenesis mechanisms, thereby providing a reference for the clinical treatment of CSR.

## 1. Introduction

Cervical spondylotic radiculopathy (CSR) is extremely common in clinical practice. Cervical radicular lesions are the most common type of cervical spondylosis, accounting for a significant proportion, ranging from 60% to 70%. It is a clinical condition caused by the deterioration of cervical disc function, degeneration, loosening of cervical joint ligaments, and compression of cervical nerve roots. The clinical manifestations primarily include neck and arm pain, numbness, limited neck movement, and a significant impact on people’s daily life and work.^[1–3]^ Due to the continuous development of social and economic levels, changes in lifestyle, work or life pressures, and other factors, the incidence of CSR has been rising year by year. Currently, in modern medicine, most interventions are carried out using drugs and surgery. However, commonly used anti-inflammatory and analgesic drugs, traction therapy, and surgical treatments not only increase treatment risks but also can lead to drug dependence, presenting a high risk. Acupuncture is particularly effective in treating cervical spondylosis and pain in other areas of the body. It is characterized by its simple procedure, high safety, and diverse treatment methods, and has gained widespread recognition in society.^[4]^

Various studies have shown that conservative treatment can achieve a certain degree of improvement, as it avoids the risks associated with surgery, making it the preferred choice for patients with CSR.^[5–7]^ In this article, we summarize the articles retrieved from PubMed (see Supplementary File 1, Supplemental Digital Content, http://links.lww.com/MD/O498 for details) and present the following summary.

## 2. Pathogenesis mechanism

### 2.1. Modern medicine’s understanding of cervical spondylotic radiculopathy

Recent advances in technology and medicine have led to significant progress in understanding CSR; however, there is still no clear consensus on its pathogenesis. According to existing literature, this paper mainly discusses 3 aspects: autoimmune factors, mechanical compression factors, and inflammatory response factors. Gertzbei’s study confirmed that patients with cervical disc herniation had higher levels of IgG and IgM in cerebrospinal fluid and serum compared to healthy individuals. The study also demonstrated the deposition of antigen-antibody complexes in the herniated disc tissue, along with increased expression of lymphocytes and macrophages. Additionally, the study confirmed that the autoimmune response theory provides theoretical support for the treatment of disc herniation.^[8]^

#### 2.1.1. Autoimmune factors

Studies have demonstrated an immune response in the nucleus pulposus of the cervical disc. When the human immune system is compromised and the cervical disc is in an unhealthy state, neuropeptides released during the immune response can trigger an inflammatory response, resulting in localized pain.^[8]^

#### 2.1.2. Mechanical compression factors

When cervical disc degeneration or herniation occurs, the surrounding soft tissues compress the cervical nerve, leading to pain.^[8]^ This mechanical compression results in the blockage of blood circulation in the neck and shoulder region. When the cervical vertebra compresses the surrounding nerve vessels, it causes local ischemia, edema, increased inflammatory factors, and accumulation of acidic metabolites in the neck and shoulder, ultimately leading to a decline in nerve root conduction, root pain, and other symptoms.^[9]^

#### 2.1.3. Inflammatory response factors

Most clinical studies have confirmed that the expression of inflammatory cytokines, such as IL-1β, TNF-α, and IL-6, is significantly increased at the site of herniated intervertebral discs. These cytokines are considered key factors in the development of radiculopathic pain in cervical radiculopathy.^[10]^ When edema or an inflammatory reaction occurs in the nerve roots of the neck and shoulder, degeneration of the intervertebral disc occurs, leading to compression and rupture of the annulus fibrosus. Inflammatory factors in the interstitial fluid then enter the spinal canal, causing pain and discomfort through infiltration and stimulation of the nerve roots.^[11]^

### 2.2. Traditional Chinese medicine (TCM) understanding of CSR with the lack of blood and Qi

In ancient Chinese medical texts, CSR was not clearly defined; however, based on clinical symptoms, it could be classified as “neck bi syndrome” or “item bi syndrome” in TCM. Although modern TCM scholars have not reached a clear conclusion on the etiology and pathogenesis of CSR, clinical signs are generally categorized into 3 categories: external pathogenic factors, internal deficiencies, and overexertion.

#### 2.2.1. Exogenous evil

The “Su Wen” chapter states, “Wind, cold, and dampness, the three Qi, appear mingled. They bind to each other and form Bi obstruction,” pointing out that the pathogenic factors of this disease are caused by the interaction of wind, cold, and dampness. Wind causes light drainage, cold leads to condensation, and dampness is heavy and viscous. These 3 evils interact, blocking the flow of Qi, resulting in neck pain and stiffness, which is referred to as Bi syndrome. If 1 is affected by the 3 evils of wind, cold, and dampness, the tongue appears pale with a white coating. Symptoms include urgent neck and shoulder pain, which worsens on rainy days, along with joint pain and muscle stiffness or numbness. The primary treatment goal is to dissipate the cold and dampness from the body and alleviate numbness and pain. The convergence of the 3 evil Qi of wind, cold, and dampness impairs the function of the 5 internal organs, and the imbalance of yin and yang creates conditions for disease development. If left untreated or improperly treated over time, the 3 evils of wind, cold, and dampness persist in the neck muscles, bones, and joints, causing blockage of the meridians. This impedes the smooth flow of Qi and blood, leading to blood stasis, and results in stiffness and weakness in the limbs and muscles. Eventually, this causes a deficiency of Qi and blood, contributing to the development of disease.

#### 2.2.2. The internal

The “Su Wen” chapter states: “Ying Qi deficiency is not benevolent, Wei Qi deficiency is not essential, and the deficiency of both Ying and Wei Qi leads to a lack of both benevolence and necessity.” When the body is weak and out of harmony, Qi and blood are lost from the skin, muscles, bones, and viscera, leading to a series of symptoms, such as pain, numbness, and other related manifestations.^[12]^ If the disease is caused by a deficiency of Qi and blood, the main manifestations include a pale tongue with a white coating, fatigue, and numbness in the fingers. The treatment primarily focuses on tonifying Qi.

Deficiency of Qi and blood in the body results in a deficiency of healthy Qi. The impaired movement of Qi leads to slow blood flow, weak circulation, and poor Qi operation, causing Qi and blood stagnation around the neck, which results in the accumulation of blood stasis and the development of CSR, characterized by a lack of Qi and blood.

#### 2.2.3. Fatigue

The “Su Wen” chapter states: “Labor depletes Qi.” Excessive fatigue and damage to the body’s vitality result in a deficiency of Qi and blood. When the neck meridians are affected, the Qi and blood in the neck become stagnant, causing localized pain, which leads to the development of nerve root-type cervical spondylosis. If the disease is caused by a deficiency of blood and Qi, the main manifestations include a dark red tongue and astringency, along with tingling in the neck and shoulders. The treatment should focus on promoting blood circulation and relieving pain. During the progression of the disease, various etiologies and pathogenesis factors can transform into each other. In the early stages of the disease, the pathogenic attack of wind, cold, and dampness, if untreated or inadequately treated, can lead to a deficiency of blood and Qi. Qi and blood are fundamental to human health, and their deficiency leads to bodily weakness, creating conditions for disease development. Such a cycle forms a vicious circle that impedes the body’s recovery. Therefore, when faced with diseases characterized by both deficiency and excess, it is essential to differentiate between deficiency and excess in treatment, so as to achieve the therapeutic goal of tonifying the deficiency and expelling the pathogenic factors.^[13]^

## 3. Mechanism

The theory of meridians and acupoints forms the theoretical basis of acupuncture treatment, while the efficacy of acupuncture serves as a practical validation of this meridian theory. Most research on the essence of meridians and the mechanisms of acupuncture is based on known structures, such as nerves, blood vessels, fascia, mast cells, and immune system components, with few significant breakthroughs. Imbalances in Yin and Yang or disharmony among the internal organs can lead to pain in the corresponding areas of the body. Acupuncture can alleviate symptoms, identify the underlying causes of pain, and restore homeostatic balance.^[14]^ This stands in stark contrast to the surgical and analgesic drug treatments commonly employed in Western medicine. This article will explore the mechanisms of acupuncture and moxibustion from 2 key aspects.

### 3.1. Anti-inflammatory theory

During inflammation, the expression of cytokines such as IL-1β, TNF-α, and IL-6 increases significantly in the herniated intervertebral disc.^[10]^ As inflammation progresses, these factors induce neuronal hyperexcitability, amplify the pain sensation, and contribute to chronic or neuropathic pain.^[14]^ Currently, the theory that acupuncture alleviates pain by modulating inflammation is widely accepted by scholars. Different acupuncture points are capable of modulating specific inflammatory cells. Studies of numerous animal models have shown that stimulation of Zusanli (ST36) can significantly alter inflammatory mediators and enhance immune function.^[14]^ The anti-inflammatory effect of this point is also mediated by the activity of the vagus nerve, TLR4/NF-κB signaling pathway, c-Kit signaling pathway, and several other mechanisms. According to Cenning Li et al’s^[15]^ evaluating the anti-inflammatory mechanism of acupuncture, acupuncture alters connective tissue, induces the secretion of various molecules at acupuncture points, and activates the NF-κB, MAPK, and ERK pathways in mast cells, fibroblasts, keratinocytes, and monocytes/macrophages, thereby supporting the validity of acupuncture’s anti-inflammatory mechanism.

### 3.2. Neurotransmitters

#### 3.2.1. Serotonin

Serotonin is commonly regarded as a neurotransmitter involved in pain relief. Both conventional acupuncture and electroacupuncture reduce pro-inflammatory cytokines in the periphery and spinal cord, along with serotonin and norepinephrine, during the analgesic process.^[16]^ Acupuncture significantly reduces the secretion of the pain mediator, 5-HT.^[17]^ The serotonin pain modulation system plays a role in the analgesic mechanism of acupuncture. In related experimental studies, Liu et al^[18]^ measured the concentrations of 5-HT and 5-HT4R in rats with chronic visceral hypersensitivity following electroacupuncture stimulation using ELISA. The results showed that electroacupuncture significantly increased pain thresholds, reduced the concentration of 5-HT, and effectively elevated the concentration of 5-HT4R. These studies clearly indicate that serotonin is involved in the analgesic effect of acupuncture. However, to achieve optimal analgesic effects, the appropriate acupuncture points must be selected.

#### 3.2.2. Opioid peptides

Supported by numerous animal studies and clinical research, acupuncture is an effective pain relief method. Electroacupuncture stimulation facilitates the release of endogenous opioid peptides, leading to significant analgesic effects.^[19]^ The endogenous opioid peptide mechanism is the most well-established neuronal mechanism in acupuncture analgesia, primarily involving 4 categories: enkephalins, endorphins, dynorphins, and nociceptin.^[20]^

## 4. Acupuncture therapies

### 4.1. Combination of acupuncture and empirical theory-guided treatment

Acupuncture treatment effectively promotes the movement of local meridians, alleviates muscle spasms, reduces numbness and pain caused by nerve compression, and aids in the recovery of nerve function. Modern medicine considers the symptoms of nerve root-type cervical spondylosis to resemble the “lack of blood and Qi” syndrome in traditional Chinese medicine (TCM), which can be treated by promoting blood circulation and alleviating pain through Qi regulation. Peng Siping employed the 3-way balanced acupuncture method to treat CSR, based on Mr. Jin Rui’s “Jin San-acupuncture” theory, a cornerstone of modern acupuncture and moxibustion. This method emphasizes channeling meridians, harmonizing Zang-Fu organs, and balancing the Yuan spirit during treatment. The treatment lasted 4 weeks, with 80 patients diagnosed with CSR and the “lack of blood and Qi” syndrome divided into 2 groups: 40 patients in the control group received conventional acupuncture, while 40 patients in the treatment group received Sandong balance acupuncture. The experimental results showed that the treatment group had a total effective rate of 95.0%, while the control group had a rate of 80.0%. Both treatments were effective, but the treatment group showed significantly better results in alleviating pain and anxiety caused by CSR.^[21]^ Zhong Zhiwei applied the theory of the “three arguments” to guide the treatment of CSR. The treatment cycle lasted 2 weeks, with 10 sessions in total. The control group received routine point selection based on the syndrome types of Qi stagnation and blood stasis, such as cervical Jiaji, Tianzhu, Fengchi, Quchi, Waiguan, and Aishi points, using quick straight needle insertion.

The observation group first performed disease differentiation, scriptural analysis, and dialectical reasoning, after which relevant points were selected. The results showed that the effective rate was 83.33% in the observation group and 60% in the control group. This indicates that both treatments are effective, but the therapeutic effect of CSR under the guidance of the “three differences” theory is superior to that of conventional acupuncture. Under the guidance of the “three theories,” acupuncture treatment for CSR with the “lack of blood and Qi” syndrome can achieve pain relief, improve cervical spine mobility, and restore normal life.^[22]^ The 2 treatment methods described above are based on conventional acupuncture combined with the empirical theories of renowned classical Chinese medicine, highlighting the positive role of innovation in integrating the foundational principles of TCM with acupuncture theory, thereby offering more effective and diversified theoretical guidance for the future treatment of CSR.

### 4.2. Acupuncture combined with pricking bloodletting and cupping treatment

The syndrome of CSR, characterized by a deficiency of blood and Qi, is primarily caused by blood stasis in the veins, leading to poor circulation, impaired blood flow, and pain. The treatment should focus on activating blood flow and dredging the meridians. “The Medical King Golden Canon” states: “Cupping draws out the stagnant blood to restore balance.” Acupuncture and cupping can significantly alleviate Qi and blood stagnation in the local meridians, facilitate the outflow of stagnant substances in the blood, reduce local edema, repair tissues, improve microcirculation, and ultimately achieve the therapeutic goal of “removing blood stasis and promoting the generation of new blood.”^[23,24]^

Zhang explored the clinical therapeutic value of acupuncture and cupping by observing, comparing, and analyzing the differences in clinical efficacy between acupuncture combined with cupping and conventional acupuncture in CSR. The study confirmed that acupuncture combined with cupping is more effective than simple acupuncture, and that the treatment process is well-accepted by patients. Grounded in the profound theory of blood and Qi deficiency, this treatment highlights the advantages of acupuncture in TCM and aligns with its holistic principles. It can fundamentally eliminate blood stasis and poor circulation, effectively alleviate discomfort symptoms in patients with CSR characterized by blood and Qi deficiency, and enhance their quality of life.^[25]^

### 4.3. Acupuncture combined with traditional medicine

The use of Chinese herbs is one of the oldest forms of remedy. In recent years, TCM have garnered more attention for pain relief due to their lower dependency and fewer side effects compared to synthetic drugs. The pressing need to identify a herbal remedy that can effectively alleviate neuropathic pain has become a top priority for researchers. Studies have demonstrated that Chinese herbal medicine offers a viable alternative for relieving and managing neuropathic pain. Many of the pathways involved in herbal analgesia exert antioxidant, anti-inflammatory, anti-apoptotic, neuroprotective, and calcium-inhibitory effects.^[26,27]^ Fu Shan, a Chinese physician from the Qing Dynasty, once stated, “If blood circulation is not promoted to remove blood stasis after a prolonged illness, how can one eliminate the stagnation of aging and break the stasis formed by chronic closure?” It is evident that the syndrome of CSR characterized by a deficiency of blood and Qi should be treated by “supplementing Qi, activating blood, promoting circulation, and relieving pain.” Experimental studies have confirmed that TCM drugs that promote blood circulation and remove blood stasis have a significant impact on the rehabilitation of CSR.^[28]^ Among these drugs, the most commonly used are *Salvia miltiorrhiza*, safflower, frankincense, myrrh, and *Ligusticum chuanxiong*. Flexible compatibility between these drugs can help reduce plasma viscosity, enhance myocardial function, promote blood circulation and relieve pain, reduce swelling, and aid in muscle recovery.^[29]^ Wang Qiaoling divided 87 patients into a control group, which received oral ibuprofen sustained-release capsules and flunarizine tablets, and a treatment group, which received oral *XiQixing Qifang* combined with acupuncture, based on the treatment protocol of the control group. After 4 weeks of treatment, clinical results showed that the effective rate of the treatment group was as high as 95.4%, significantly higher than the 81.4% effective rate observed in the control group.^[30]^ Wang Yaqin discussed the use of *Shentong Zhuyu Decoction* combined with acupuncture to treat the syndrome of CSR with a lack of blood and Qi. She divided 60 patients into 2 groups: a control group that received acupuncture treatment in addition to conventional drug therapy, and an observation group that received *Shentong Zhuyu Decoction* alongside the treatment in the control group. By comparing the clinical effects of the 2 groups, the observation group showed a significantly higher total effective rate than the control group (*P* < .05), suggesting that the combination of Chinese medicine and acupuncture can effectively promote blood circulation and reduce local edema.^[31]^ Zhang Wei combined *Qiang Huosengshi Decoction* with acupuncture and randomly divided 41 patients into treatment and control groups. The treatment group received both acupuncture and *Qiang Huosengshi Decoction*, while the control group received only the decoction. Patients with the lack of blood and Qi syndrome were treated with *Duhuo Jizhi Decoction*, while those with wind-cold obstruction syndrome received *Qiangsheng Shengshi Decoction*. The treatment was administered once per day for 7 days, with a total of 3 courses. The final results showed that the control group had an efficacy rate of 70%, while the treatment group had an efficacy rate of 90.7%. The *Duhuo*, *Mulberry Jishi*, and *Chuanxiong* herbs used in the formulation were combined with acupuncture points such as *Quchi*, *Hegu*, *Houxi*, *Zhongzhu*, and *LiQi*, fully demonstrating their effects of dredging meridians, dispersing wind, dispelling cold, promoting blood circulation, and removing blood stasis.^[32]^

### 4.4. Acupuncture combined with point injection treatment

Acupoint injection combined with acupuncture is a commonly used clinical treatment approach. Guided by the TCM theory of meridian acupoints, this technique involves injecting therapeutic solutions into reactionary points followed by regular acupuncture in the affected area. This approach fully integrates the influence of acupuncture on Qi, the therapeutic effect of drugs, the conductivity of meridians, and the specificity of acupoint selection. It offers advantages such as ease of operation, absence of side effects, good patient acceptance, ease of adherence, and low cost. This is a clinically safe and effective treatment approach.^[33]^ The combination of traditional Chinese and Western medicine therapies leads to better clinical outcomes. Yajie^[33]^ included 78 patients with cervical radiculopathy, treating them with acupuncture combined with acupoint injection. The experiment’s conclusion showed that the total effective rate in the observation group treated with vitamin B_12_ combined with acupuncture was 94.87%, significantly higher than the cure rate of 66.67% in the control group, which received acupuncture alone. The zVAS score of the observation group was significantly lower than that of the control group. In the comparison of VAS scores, the observation group had a significantly lower score, while the results for SF-36 quality of life function scores were the opposite.^[34]^ Jinsuo^[34]^ divided 70 patients into 2 groups, treating 1 group with Danshen injection combined with acupuncture for cervical spondylosis with a syndrome of lack of blood and Qi over the course of 1 month. The treatment group showed a significant advantage of 84.9% over the control group, suggesting that acupuncture combined with acupoint injection offers an effective clinical therapeutic approach for treating arthralgia, and provides valuable insight for treating other types of cervical spondylosis with a syndrome of lack of blood and Qi_._^[35]^

## 5. Summary

In summary, acupuncture offers several advantages in treating the Qi-stagnation and blood-stasis syndrome of nerve root type cervical spondylosis, including rapid effects, minimal side effects, and ease of application. When combined with other treatment methods, acupuncture shows enhanced therapeutic effects and has been widely studied in clinical research. As stated in the “Inner Classic,” “When needles are used, deficiency leads to reality, fullness leads to thinning, stasis leads to elimination, and evil victory leads to deficiency.” Acupuncture and moxibustion therapy, by addressing the clinical signs of patients with the lack of blood and Qi syndrome, tonifies and replenishes deficiency, promotes blood circulation, and ensures smooth Qi and blood flow, achieving the balance of Yin and Yang. In treating CSR with a lack of blood and Qi syndrome, combining acupuncture with cupping, Chinese herbal medicine, acupoint injection, traction, and massage can significantly enhance clinical outcomes and alleviate patient pain. Overall, acupuncture provides a safe and effective alternative therapy, offering new hope for patients suffering from pain. By integrating various treatment methods, the benefits of each can be fully utilized to improve treatment efficacy, alleviate symptoms, and enhance patients’ quality of life. During treatment, objective assessment tools such as neuromuscular function evaluation and imaging should be used to quantify therapeutic effects. This enables more precise monitoring of patient progress and provides a more scientific basis for treatment. However, the limitations of acupuncture should not be overlooked in clinical practice. Improper acupuncture technique can cause local capillary rupture, acupoint infections, and other side effects, and may even result in temporary shock in patients who experience dizziness. Therefore, it is essential to consult medical professionals before treatment to avoid such complications. Acupuncture combined with other treatments can significantly improve cervical radiculopathy, reducing pain and alleviating symptoms. The combination of acupuncture and TCM effectively enhances the overall therapeutic rate and improves symptoms in patients with cervical radiculopathy. Acupuncture combined with bloodletting therapy proves highly effective, improving local numbness and pain, enhancing microcirculation, and reducing blood viscosity. Future research should focus on developing personalized treatment plans based on patients’ specific conditions and physical status, tailoring acupuncture combined with other treatments for improved efficacy.

## Author contributions

**Writing – original draft:** Furong Xue.

**Writing – review & editing:** Weiguo Liu.

## Supplementary Material


