# Supplementary material for: Research progress on acupuncture intervention for cervical spondylotic radiculopathy with Qi stagnation and blood stasis syndrome: A review
Source: Medicine (Baltimore). 2025 Apr 4;104(14):e41837. doi: 10.1097/MD.0000000000041837 (PMC11977727; doi:10.1097/MD.0000000000041837)
Supplement: Supplementary file 1 [file medi-104-e41837-s001.docx]

Supplementary File 1:The searching strategies and databases for references.

| Search number | Query | Search Details | Results |
| --- | --- | --- | --- |
|  | (("Radiculopathy"[Mesh]) OR ((((((((((((((Radiculopathies[Title/Abstract]) OR (Radiculitis[Title/Abstract])) OR (Radiculitides[Title/Abstract])) OR (Nerve Root Inflammation[Title/Abstract])) OR (Inflammation, Nerve Root[Title/Abstract])) OR (Nerve Root Inflammations[Title/Abstract])) OR (Nerve Root Compression[Title/Abstract])) OR (Compression, Nerve Root[Title/Abstract])) OR (Compressions, Nerve Root[Title/Abstract])) OR (Nerve Root Compressions[Title/Abstract])) OR (Nerve Root Disorder[Title/Abstract])) OR (Nerve Root Disorders[Title/Abstract])) OR (Cervical Radiculopathies[Title/Abstract])) OR (Cervical Radiculopathy[Title/Abstract]))) AND (("Acupuncture Therapy"[Mesh]) OR ((((((((Acupuncture Treatment[Title/Abstract]) OR (Acupuncture Treatments[Title/Abstract])) OR (Treatment, Acupuncture[Title/Abstract])) OR (Therapy, Acupuncture[Title/Abstract])) OR (Pharmacoacupuncture Treatment[Title/Abstract])) OR (Treatment, Pharmacoacupuncture[Title/Abstract])) OR (Pharmacoacupuncture Therapy[Title/Abstract])) OR (Therapy, Pharmacoacupuncture[Title/Abstract]))) | ("Radiculopathy"[MeSH Terms] OR ("Radiculopathies"[Title/Abstract] OR "Radiculitis"[Title/Abstract] OR "Radiculitides"[Title/Abstract] OR "nerve root inflammation"[Title/Abstract] OR (("Inflammation"[MeSH Terms] OR "Inflammation"[All Fields] OR "Inflammations"[All Fields] OR "inflammation s"[All Fields]) AND "nerve root"[Title/Abstract]) OR "nerve root inflammations"[Title/Abstract] OR "nerve root compression"[Title/Abstract] OR "compression nerve root"[Title/Abstract] OR (("compress"[All Fields] OR "compressed"[All Fields] OR "compresses"[All Fields] OR "compressibilities"[All Fields] OR "compressibility"[All Fields] OR "compressible"[All Fields] OR "compressing"[All Fields] OR "Compression"[All Fields] OR "compression s"[All Fields] OR "Compressions"[All Fields] OR "compressive"[All Fields] OR "compressively"[All Fields]) AND "nerve root"[Title/Abstract]) OR "nerve root compressions"[Title/Abstract] OR "nerve root disorder"[Title/Abstract] OR "nerve root disorders"[Title/Abstract] OR "cervical radiculopathies"[Title/Abstract] OR "cervical radiculopathy"[Title/Abstract])) AND ("Acupuncture Therapy"[MeSH Terms] OR ("acupuncture treatment"[Title/Abstract] OR "acupuncture treatments"[Title/Abstract] OR "treatment acupuncture"[Title/Abstract] OR "therapy acupuncture"[Title/Abstract] OR "pharmacoacupuncture treatment"[Title/Abstract] OR (("therapeutics"[MeSH Terms] OR "therapeutics"[All Fields] OR "Treatments"[All Fields] OR "Therapy"[MeSH Subheading] OR "Therapy"[All Fields] OR "Treatment"[All Fields] OR "treatment s"[All Fields]) AND "Pharmacoacupuncture"[Title/Abstract]) OR "pharmacoacupuncture therapy"[Title/Abstract] OR (("therapeutics"[MeSH Terms] OR "therapeutics"[All Fields] OR "therapies"[All Fields] OR "Therapy"[MeSH Subheading] OR "Therapy"[All Fields] OR "therapy s"[All Fields] OR "therapys"[All Fields]) AND "Pharmacoacupuncture"[Title/Abstract]))) | 87 |
| 9 | ("Acupuncture Therapy"[Mesh]) OR ((((((((Acupuncture Treatment[Title/Abstract]) OR (Acupuncture Treatments[Title/Abstract])) OR (Treatment, Acupuncture[Title/Abstract])) OR (Therapy, Acupuncture[Title/Abstract])) OR (Pharmacoacupuncture Treatment[Title/Abstract])) OR (Treatment, Pharmacoacupuncture[Title/Abstract])) OR (Pharmacoacupuncture Therapy[Title/Abstract])) OR (Therapy, Pharmacoacupuncture[Title/Abstract])) | "Acupuncture Therapy"[MeSH Terms] OR ("acupuncture treatment"[Title/Abstract] OR "acupuncture treatments"[Title/Abstract] OR "treatment acupuncture"[Title/Abstract] OR "therapy acupuncture"[Title/Abstract] OR "pharmacoacupuncture treatment"[Title/Abstract] OR (("therapeutics"[MeSH Terms] OR "therapeutics"[All Fields] OR "Treatments"[All Fields] OR "Therapy"[MeSH Subheading] OR "Therapy"[All Fields] OR "Treatment"[All Fields] OR "treatment s"[All Fields]) AND "Pharmacoacupuncture"[Title/Abstract]) OR "pharmacoacupuncture therapy"[Title/Abstract] OR (("therapeutics"[MeSH Terms] OR "therapeutics"[All Fields] OR "therapies"[All Fields] OR "Therapy"[MeSH Subheading] OR "Therapy"[All Fields] OR "therapy s"[All Fields] OR "therapys"[All Fields]) AND "Pharmacoacupuncture"[Title/Abstract])) | 31,660 |
| 8 | ("Radiculopathy"[Mesh]) OR ((((((((((((((Radiculopathies[Title/Abstract]) OR (Radiculitis[Title/Abstract])) OR (Radiculitides[Title/Abstract])) OR (Nerve Root Inflammation[Title/Abstract])) OR (Inflammation, Nerve Root[Title/Abstract])) OR (Nerve Root Inflammations[Title/Abstract])) OR (Nerve Root Compression[Title/Abstract])) OR (Compression, Nerve Root[Title/Abstract])) OR (Compressions, Nerve Root[Title/Abstract])) OR (Nerve Root Compressions[Title/Abstract])) OR (Nerve Root Disorder[Title/Abstract])) OR (Nerve Root Disorders[Title/Abstract])) OR (Cervical Radiculopathies[Title/Abstract])) OR (Cervical Radiculopathy[Title/Abstract])) | "Radiculopathy"[MeSH Terms] OR ("Radiculopathies"[Title/Abstract] OR "Radiculitis"[Title/Abstract] OR "Radiculitides"[Title/Abstract] OR "nerve root inflammation"[Title/Abstract] OR (("Inflammation"[MeSH Terms] OR "Inflammation"[All Fields] OR "Inflammations"[All Fields] OR "inflammation s"[All Fields]) AND "nerve root"[Title/Abstract]) OR "nerve root inflammations"[Title/Abstract] OR "nerve root compression"[Title/Abstract] OR "compression nerve root"[Title/Abstract] OR (("compress"[All Fields] OR "compressed"[All Fields] OR "compresses"[All Fields] OR "compressibilities"[All Fields] OR "compressibility"[All Fields] OR "compressible"[All Fields] OR "compressing"[All Fields] OR "Compression"[All Fields] OR "compression s"[All Fields] OR "Compressions"[All Fields] OR "compressive"[All Fields] OR "compressively"[All Fields]) AND "nerve root"[Title/Abstract]) OR "nerve root compressions"[Title/Abstract] OR "nerve root disorder"[Title/Abstract] OR "nerve root disorders"[Title/Abstract] OR "cervical radiculopathies"[Title/Abstract] OR "cervical radiculopathy"[Title/Abstract]) | 10,112 |
| 7 | (qi stagnation and blood stasis syndrome[Title/Abstract]) OR (blood stasis[Title/Abstract]) | "qi stagnation and blood stasis syndrome"[Title/Abstract] OR "blood stasis"[Title/Abstract] | 2,687 |
| 6 | blood stasis[Title/Abstract] | "blood stasis"[Title/Abstract] | 2,687 |
| 5 | qi stagnation and blood stasis syndrome[Title/Abstract] | "qi stagnation and blood stasis syndrome"[Title/Abstract] | 26 |
| 4 | (((((((Acupuncture Treatment[Title/Abstract]) OR (Acupuncture Treatments[Title/Abstract])) OR (Treatment, Acupuncture[Title/Abstract])) OR (Therapy, Acupuncture[Title/Abstract])) OR (Pharmacoacupuncture Treatment[Title/Abstract])) OR (Treatment, Pharmacoacupuncture[Title/Abstract])) OR (Pharmacoacupuncture Therapy[Title/Abstract])) OR (Therapy, Pharmacoacupuncture[Title/Abstract]) | "acupuncture treatment"[Title/Abstract] OR "acupuncture treatments"[Title/Abstract] OR "treatment acupuncture"[Title/Abstract] OR "therapy acupuncture"[Title/Abstract] OR "pharmacoacupuncture treatment"[Title/Abstract] OR (("therapeutics"[MeSH Terms] OR "therapeutics"[All Fields] OR "Treatments"[All Fields] OR "Therapy"[MeSH Subheading] OR "Therapy"[All Fields] OR "Treatment"[All Fields] OR "treatment s"[All Fields]) AND "Pharmacoacupuncture"[Title/Abstract]) OR "pharmacoacupuncture therapy"[Title/Abstract] OR (("therapeutics"[MeSH Terms] OR "therapeutics"[All Fields] OR "therapies"[All Fields] OR "Therapy"[MeSH Subheading] OR "Therapy"[All Fields] OR "therapy s"[All Fields] OR "therapys"[All Fields]) AND "Pharmacoacupuncture"[Title/Abstract]) | 4,256 |
| 3 | "Acupuncture Therapy"[Mesh] | "Acupuncture Therapy"[MeSH Terms] | 30,543 |
| 2 | (((((((((((((Radiculopathies[Title/Abstract]) OR (Radiculitis[Title/Abstract])) OR (Radiculitides[Title/Abstract])) OR (Nerve Root Inflammation[Title/Abstract])) OR (Inflammation, Nerve Root[Title/Abstract])) OR (Nerve Root Inflammations[Title/Abstract])) OR (Nerve Root Compression[Title/Abstract])) OR (Compression, Nerve Root[Title/Abstract])) OR (Compressions, Nerve Root[Title/Abstract])) OR (Nerve Root Compressions[Title/Abstract])) OR (Nerve Root Disorder[Title/Abstract])) OR (Nerve Root Disorders[Title/Abstract])) OR (Cervical Radiculopathies[Title/Abstract])) OR (Cervical Radiculopathy[Title/Abstract]) | "Radiculopathies"[Title/Abstract] OR "Radiculitis"[Title/Abstract] OR "Radiculitides"[Title/Abstract] OR "nerve root inflammation"[Title/Abstract] OR (("Inflammation"[MeSH Terms] OR "Inflammation"[All Fields] OR "Inflammations"[All Fields] OR "inflammation s"[All Fields]) AND "nerve root"[Title/Abstract]) OR "nerve root inflammations"[Title/Abstract] OR "nerve root compression"[Title/Abstract] OR "compression nerve root"[Title/Abstract] OR (("compress"[All Fields] OR "compressed"[All Fields] OR "compresses"[All Fields] OR "compressibilities"[All Fields] OR "compressibility"[All Fields] OR "compressible"[All Fields] OR "compressing"[All Fields] OR "Compression"[All Fields] OR "compression s"[All Fields] OR "Compressions"[All Fields] OR "compressive"[All Fields] OR "compressively"[All Fields]) AND "nerve root"[Title/Abstract]) OR "nerve root compressions"[Title/Abstract] OR "nerve root disorder"[Title/Abstract] OR "nerve root disorders"[Title/Abstract] OR "cervical radiculopathies"[Title/Abstract] OR "cervical radiculopathy"[Title/Abstract] | 6,004 |
| 1 | "Radiculopathy"[Mesh] | "Radiculopathy"[MeSH Terms] | 6,034 |
